# Evaluating compliance and applicability of postpartum pessary use for preventing and treating pelvic floor dysfunction: a prospective multicenter study

**DOI:** 10.1007/s00404-023-07075-9

**Published:** 2023-05-21

**Authors:** Brenda Kiefner, Frank Schwab, Madeleine Kuppinger, Anna Nacke, Ute Kelkenberg, Sabine Schütze, Franziska Berger, Anna Lindner, Lars Hellmeyer, Wolfgang Janni, Melanie Metz, Miriam Deniz

**Affiliations:** 1 Universitatsklinikum Ulm, Ulm, Germany; 2 Charité Universitätsmedizin Berlin, Institute of Hygiene and Environmental Medicine, Berlin, Germany; 3 Vivantes Klinikum Friedrichshain, Department of Obstetrics, Berlin, Germany; 4 Klinikum Bielefeld, Departement of Obstetrics and Gynecology, Bielefeld, Germany

**Keywords:** Pelvic floor dysfunction, Postpartum, Pessary therapy, Prevention, Compliance

## Abstract

**Purpose:**

Pelvic floor disorders are common and associated with pregnancy and childbirth. For restitution of pelvic floor connective tissue and thereby therapy of postpartum pelvic organ prolapse and stress urinary incontinence, the Restifem^®^ pessary is approved. It supports the anterior vaginal wall behind the symphysis, the lateral sulci and the sacro-uterine ligaments and stabilises the connective tissue. We evaluated the compliance and applicability of Restifem^®^ use in women postpartum in a preventive and therapeutic approach.

**Methods:**

Restifem^®^ pessary was handed out to 857 women. Six weeks after birth, they started the pessary use. After 8 weeks, 3 and 6 months postpartum, women received a questionnaire via online survey for evaluation of pessary applicability and efficacy.

**Results:**

After 8 weeks, 209 women answered the questionnaire. 119 women used the pessary. Common problems were discomfort, pain and the pessary use was to circuitous. Vaginal infections were rare. After 3 months, 85 women and after 6 months, 38 women still used the pessary. 3 months postpartum, 94% of women with POP, 72% of women with UI and 66% of women with OAB stated to have an improvement of their symptoms using the pessary. 88% women without any disorder felt an improvement of stability.

**Conclusions:**

Use of the Restifem^®^ pessary in the postpartum period is feasible and accompanied with less complications. It reduces POP and UI and leads to an increased sense of stability. So, Restifem^®^ pessary can be offered to women postpartum to improve pelvic floor dysfunction.

**Supplementary Information:**

The online version contains supplementary material available at 10.1007/s00404-023-07075-9.

## What does this study add to the clinical work

**Table:** 

This is the first prospective study evaluating postpartum use of the Restifem^®^ pessary, especially developed for pelvic floor restitution after childbirth. Pessary use 6 weeks postpartum is feasible and accompanied with reduction of pelvic floor dysfunction.	

## Introduction

**Fig. 1:**
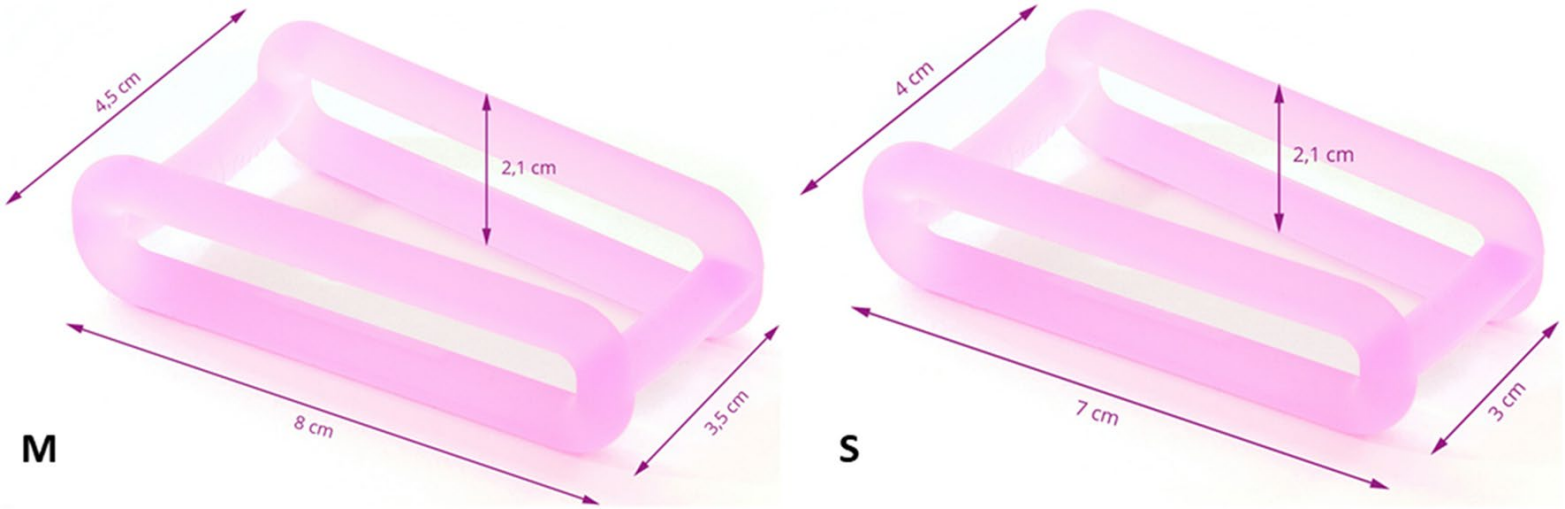
The Restifem pessary© in size M and S

Pelvic floor disorders as stress urinary incontinence (SUI), pelvic organ prolapse (POP) and overactive bladder (OAB) are prevalent and associated with pregnancy and childbirth [1, 2]. The causes of postpartum pelvic floor dysfunctions include injuries to muscular, neurogenic and connective tissue structures [3–5]. The recovery of the pelvic floor muscles is a dynamic process. Several ultrasound studies indicate that the contractility of the puborectalis muscle mostly recovers by around 12 weeks postpartum, but with a persistent increased distensibility of the hiatus [6, 7]. Pelvic floor muscle training (PFMT) is widely recommended to women after childbirth to address the muscular structures, although there is no or low-quality evidence for therapy and preventing urinary incontinence or POP [8–10]. Electrostimulation therapy can also be used to improve the nerve function in women with urinary incontinence [11]. Besides PFMT and electrostimulation, pessaries are widely used as conservative treatment option for POP and SUI [12, 13]. For rehabilitation of the connective tissue after muscle and ligamentous injuries, orthopaedic therapy often involves early mobilisation and use of stabilising medical devices [14]. By now, there is no therapeutic approach to address the pelvic floor tissue regeneration after childbirth. There are only two pilot studies investigating the effect of an early postpartum pessary treatment on women with POP or UI [15, 16]. Both studies showed that pessary therapy postpartum was feasible, but they involved small sample sizes (16 women for Baessler et al. and 15 women for Lange et al. in treatment group). Baessler et al. found that a therapy with a vaginal ring pessary might reduce POP 6 weeks after birth, whilst Lange et al. showed that women with urinary incontinence (UI) benefited from a cube pessary therapy. In Germany, the Restifem^®^ (Restitutio feminina) pessary is approved for therapy of especially postpartum UI and POP (Fig. 1). Due to its longitudinal form and the two transverse brackets, the Restifem^®^ assists all three levels of support [17, 18] the lateral vaginal wall (Level II), the uterus (Level I) and sub-urethral anterior vaginal wall (Level III). The Restifem^®^ itself rests against the symphysis and should therefore relieve the levator ani muscle and the connective tissue [19]. The primary outcome of this prospective, multicentre study was to evaluate the willingness and compliance of postpartum women with and without pelvic floor disorders regarding the use of the Restifem^®^ pessary in a therapeutic but furthermore especially in a preventive manner. Secondary outcomes were to detect applicability, main problems and complications within pessary use as well as the impact on symptoms of pelvic floor dysfunction postpartum.

## Methods

The study was approved by two ethics committees (University of Ulm (No 90/21) and Ärztekammer Berlin (No Eth-04/21)) and registered at the DRKS (DRKS00024733).

### Study population

The study was conducted in three tertiary care hospitals in Germany in the year 2021. Women were asked at the first or second day after delivery to take part in the study. Inclusion criterion was an age over 18 years old. Women were recruited independently of their parity, mode of delivery and third- or fourth-degree perineal tear. Exclusion criteria were women not in command of the German language, not able to read and understand the information sheets, not willing to take part in the study, with inflammation of the vulva or vagina, with intolerance of silicone and not able to insert and remove the pessary. 857 women were enrolled and gave written informed consent. The Restifem^®^ pessary was handed out directly to these women at enrolment (after caesarean section “small” Restifem^®^ and after vaginal “medium” Restifem^®^ pessary). To contact the study team in case of questions or complaints regarding the Restifem^®^ application, women received a study-specific e-mail address. After 6 weeks postpartum study, participants were prompted per e-mail to start wearing the Restifem^®^ pessary for 3–6 months as described in the instruction form and video.

### Restifem^®^

The Restifem^®^ pessary consists of silicone and was developed especially for women postpartum to support the connective tissue of the pelvic floor (Fig. 1). It is approved as medical device (class 2a, European Medical Device Directive 93/42/EWG) since 2019 for women with SUI and POP after delivery. It is recommended to use the pessary after ending of lochia for at least 3–6 months.

### Online survey

We used the LimeSurvey^®^ Software versions 3.27.4 which complies with data protection regulations. Participants’ email addresses and delivery dates were recorded in the LimeSurvey^®^ database. 6 weeks after birth, participants were automatically reminded via e-mail to start using the Restifem^®^ pessary and were provided a link to an instructional video. After 8 weeks, both 3- and 6-month postpartum participants received questionnaires via online survey to evaluate the willingness and compliance of pessary use, applicability, difficulties and efficacy of the pessary. In case, women did not answer the questionnaire, an automatically generated reminder was sent after 1 week via LimeSurvey^®^. If women still did not answer, we additionally contacted these women once per e-mail directly. Data were exported from the LimeSurvey^®^ data base in Excel after finalization of the study for further analysis.

### Questionnaire

The questionnaire surveyed 30 questions (Supplemental Fig. 1). Women were asked whether they still use the pessary (yes–no–yes, after solving problems) about the frequency of their use of the pessary (8 h a day—less than 8 h a day, or sometimes) or whether they use it willingly. If the women reported not using the pessary at all, or using it irregularly or unwillingly, they were asked about potential reasons, such as pain, discomfort, time intensity or difficulties with micturition or defecation. The understandability of the instruction sheet and video was also assessed. Several questions focussed on the handling of the pessary itself, including the correct size, ease of insertion, removal and positioning, moistening the pessary, comfort during extended wear and any necessary adjustments. Women were asked about their experiences of pressure, problems with micturition or defecation, increased vaginal fluor and vaginal infections due to pessary use. Further, the survey investigated the impact of the pessary on pelvic floor dysfunction symptoms (urinary incontinence, pelvic organ prolapse, overactive bladder and sexual function). Finally, the women were asked to rate their overall satisfaction with the pessary on a scale from 0 to 100 [20].

## Results

### Willingness in participation and compliance

**Fig. 2:**
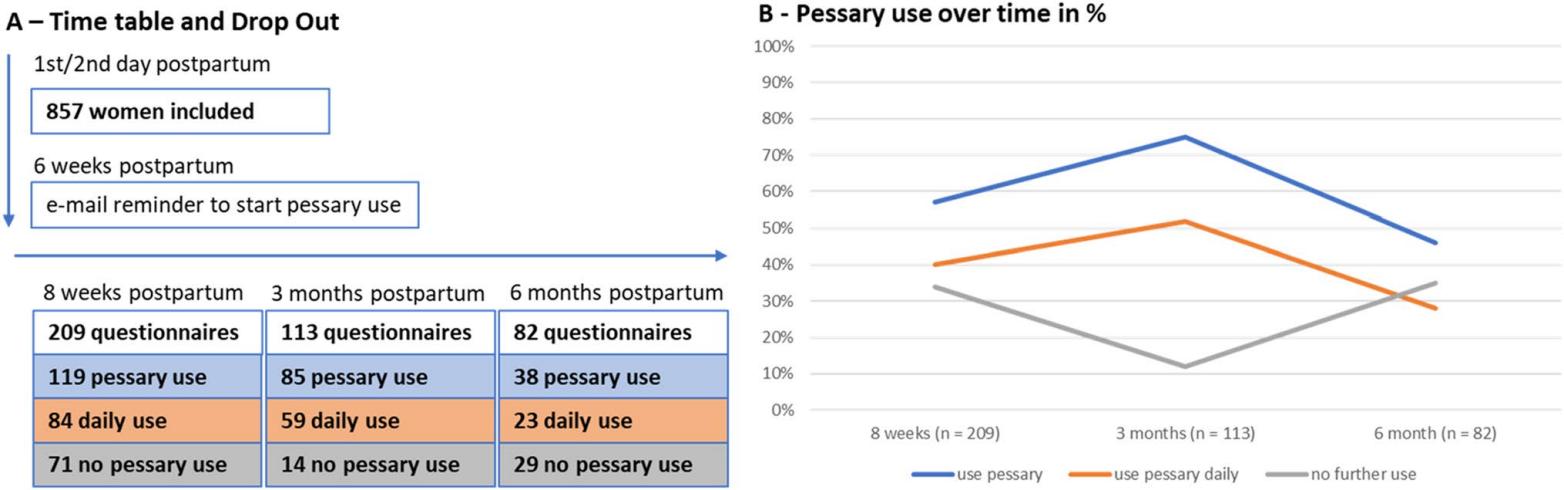
**A** Time table of the study flow and return rate of evaluable questionnaires. **B** Percentage of women using the pessary, using the pessary daily and not using the pessary over time

In one centre, we documented the proportion of women who were asked to participate. Out of 710 eligible women, 640 (90%) were interested to use the pessary. 857 women were initially enrolled in the study, all three study centres combined. Eight weeks postpartum, 273 women (31.9%) made use of the online survey and 209 (24.4%) questionnaires were filled out adequately. Out of these 209 women (100%), 119 stated to use the pessary (56.9%), thereof, 84 women on a daily basis (40.2%). 17 women (8.1%) wanted to use it after resolving problems, 71 women (34%) did not want to use the pessary any further. Three months postpartum, 199 women (23.2%) responded to the online survey and still 113 (13.2%) questionnaires were filled out adequately. Out of these 113 (100%) women, 85 stated to use the pessary (75.2%), thereof, 59 women on a daily basis (52.2%). 11 women (9.7%) wanted to use it after resolving problems, 14 women (12.4%) did not want to use the pessary any further. Six months postpartum, 146 women (17.0%) answered in the online survey and 82 (9.6%) questionnaires were filled out adequately. Out of these 82 (100%) women, 38 stated to use the pessary (46.3%), thereof, 23 women used it on a daily basis (28.0%). Seven women (8.5%) wanted to use it after resolving problems, 29 women (35.4%) did not want to use the pessary any further. These results are shown in Fig. 2A, B.

### Reasons for non-compliance and difficulties

Eight weeks postpartum, 71 women (out of 209 who answered the questionnaire) stated that they would not like to use the pessary anymore. The given reasons by these women were discomfort (63%), pain (21%) and that use was difficult (44%) or time-consuming (32%)—multiple selection was possible. Problems with micturition and defecation whilst using the pessary were not stated.

### Applicability after 2 weeks of use

Thirty-five women (25.4%) using the pessary stated that the pessary was too big. In these cases, women received a “small” pessary by mail. After 3 months, none of the women complained, that the pessary was still too big. Less than 5% of women described the instruction manual and instruction video as insufficient. Moreover, less than 5% of women required help from her midwife or physician. After 2 weeks of use, 60% of women stated that the insertion of the pessary was easy, 37% described the insertion as quite difficult and less than 2% as very difficult. 55% of women moistened the pessary with water before insertion, 4.3% used petroleum jelly, 1.5% lubricant and 1.5% hormonal cream. Removing the pessary was estimated to be easy by 83.3% and difficult by 7.2% of women.

### Compliance and inconvenience

Over time, we found no severe adverse events. None of the women consulted us about erosions or infections. In the questionnaire, altogether 11 times feeling of a vaginal infection was declared in relation to the pessary use over the entire time.

Most women used the pessary on a daily basis (Table 1). Especially between 8 weeks and 3 months, more than 50% of women made use of it every day. This proportion changed over 6 months, where only 23 women (28%) still used the pessary daily. The reasons why women did not insert the pessary often are shown in Table 1 (several answers possible).

**Table 1:** Pessary use and inconvenience

	8 weeks (*n* = 138)	3 months (*n* = 113)	6 months (*n* = 82)
How often do you wear the pessary?			
Daily—more than 8 h	53 (38%)	41 (36%)	14 (17%)
Daily less than 8 h	31 (23%)	18 (16%)	9 (11%)
Sometimes	36 (26%)	37 (33%)	27 (33%)
Never	3 (2%)	2 (2%)	0 (0%)
Missing	15 (11%)	15 (13%)	32 (39%)
Why don’t you wear the pessary often?			
Uncomfortable	11 (8%)	11 (10%)	5 (6%)
Painful	6 (4%)	2 (2%)	1 (1%)
Handling difficult	8 (6%)	8 (7%)	6 (7%)
Time-consuming	8 (6%)	14 (12%)	7 (9%)
Feeling of pressure			
Never	41 (30%)	30 (27%)	21 (26%)
Whilst walking	29 (21%)	17 (15%)	7 (9%)
When sitting	23 (17%)	16 (14%)	5 (6%)
At rest	4 (3%)	2 (2%)	0 (0%)
After defecation	45 (33%)	37 (33%)	21 (26%)
After urination	28 (20%)	15 (13%)	6 (7%)
When changing position	22 (16%)	15 (13%)	3 (4%)
Need to correct position			
Never	45 (33%)	46 (41%)	29 (35%)
Whilst walking	44 (32%)	14 (12%)	5 (6%)
When sitting	26 (19%)	18 (16%)	5 (6%)
At rest	7 (5%)	2 (2%)	1 (1%)
After defecation	29 (21%)	25 (22%)	14 (17%)
After urination	16 (12%)	14 (12%)	4 (5%)
When changing position	24 (17%)	13 (12%)	6 (7%)
Decreasing wearing comfort			
Never	46 (33%)	53 (47%)	29 (35%)
After 4 h	26 (19%)	15 (13%)	10 (12%)
After 8 h	23 (17%)	19 (17%)	5 (6%)
After 12 h	8 (6%)	6 (5%)	7 (9%)
Missing	35 (25%)	20 (18%)	31 (38%)

Total number and percentage of answers regarding questions about frequency of pessary use and inconvenience at the three time points. Multiple answers were possible

### Effect on pelvic floor symptoms and satisfaction

The proportion of women with pelvic floor disorders and the effect of the pessary use on these symptoms are shown in Table 2. Symptoms of pelvic organ prolapse were found in 23% at 8 weeks, 31% at 3 months and 18% at 6 months (Table 2). Most of women stating to have POP symptoms noticed at least a little to sometimes great improvement in symptoms using the pessary (Table 2). Urinary incontinence was found in 25% at 8 weeks, in 20% at 3 months and 16% at 6 months. Symptoms of an overactive bladder were stated by 26% at 8 weeks, 26% at 3 months and 17% at 6 months (Table 2).

**Table 2:** Pessary use and influence on symptoms

	8 weeks (*n* = 138)	3 months (*n* = 113)	6 months (*n* = 82)
Does wearing the pessary reduce problems?			
Pelvic organ prolapse			
Have no symptoms	82 (59%)	56 (50%)	35 (43%)
Not at all	3 (2%)	2 (2%)	0 (0%)
A little	18 (13%)	11 (10%)	7 (9%)
Quite	9 (7%)	17 (15%)	4 (5%)
Very	2 (1%)	5 (4%)	3 (4%)
Missig	24 (18%)	22 (19%)	33 (40%)
Urinary incontinence			
Have no symptoms	82 (59%)	69 (61%)	34 (41%)
Not at all	9 (7%)	6 (5%)	5 (6%)
A little	15 (11%)	12 (11%)	7 (9%)
Quite	8 (6%)	4 (4%)	1 (1%)
Very	2 (1%)	0 (0%)	0 (0%)
Missig	22 (16%)	22 (19%)	35 (43%)
Overactive bladder			
Have no symptoms	75 (54%)	58 (51%)	31 (38%)
Not at all	18 (13%)	10 (9%)	6 (7%)
A little	14 (10%)	12 (11%)	7 (9%)
Quite	3 (2%)	6 (5%)	2 (2%)
Very	1 (1%)	1 (1%)	0 (0%)
Missig	27 (20%)	26 (23%)	36 (44%)
Does the pessary improve pelvic floor stability?			
Very	8 (6%)	9 (8%)	6 (7%)
Quite	26 (19%)	23 (20%)	16 (20%)
A little	49 (36%)	37 (33%)	15 (18%)
Not at all	9 (6. %)	9 (8%)	6 (7%)
Worse	0 (0%)	0 (0%)	0 (0%)
Missing	46 (33%)	35 (31%)	39 (48%)

Total number and percentage of answers regarding questions about pelvic floor disorder symptoms and influence of pessary use. Multiple answers were possible

**Fig. 3:**
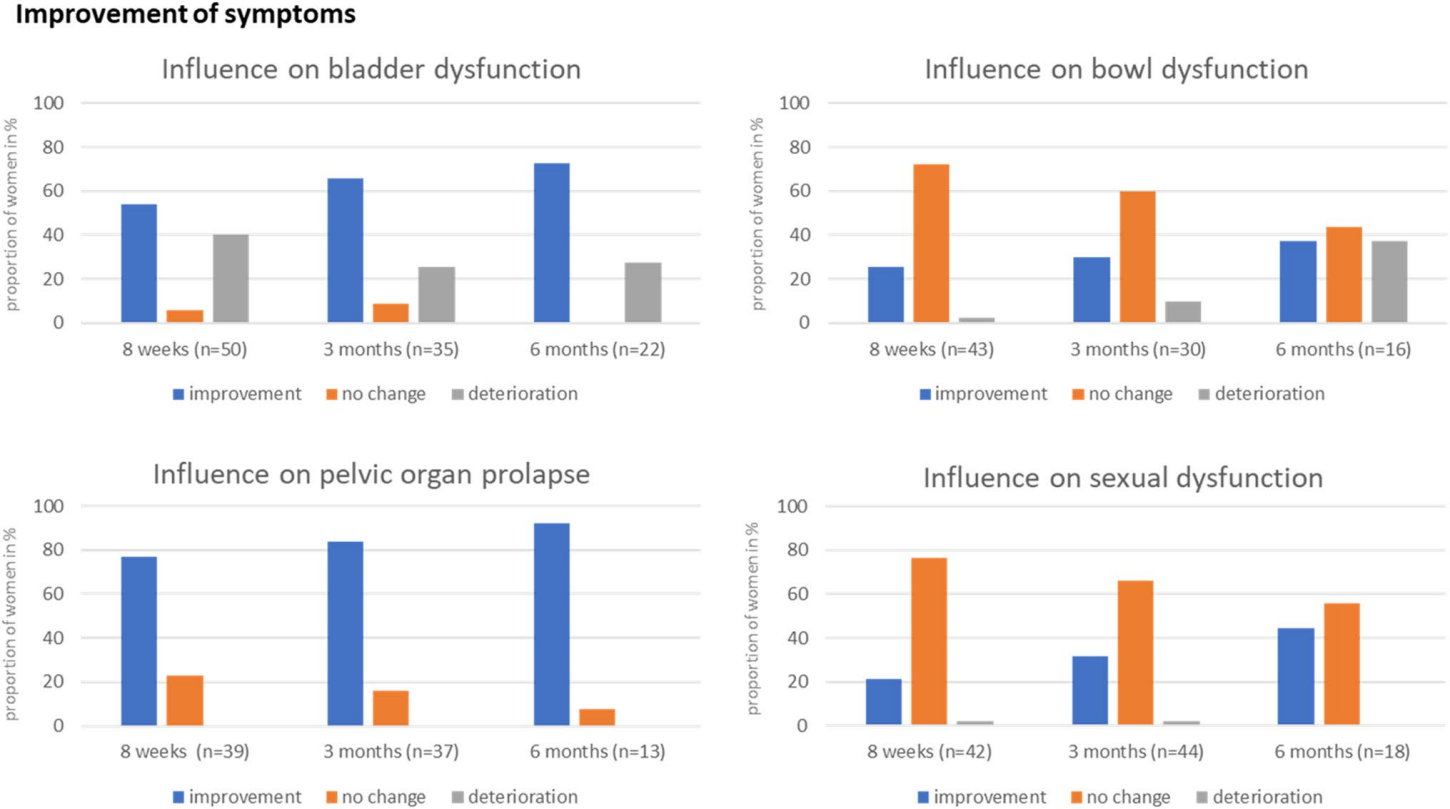
Percentage of improvement, deterioration and unchanged symptoms in women with bladder dysfunction, bowl dysfunction, pelvic organ prolapse or sexual dysfunction at the three time points

Figure 3 shows the results regarding the questions about “Overall satisfaction” in the questionnaire (Supplemental Fig. 1). Here, we determined a great and slight improvement as “improvement” as well as some and great deterioration as “deterioration”.

**Fig. 4:**
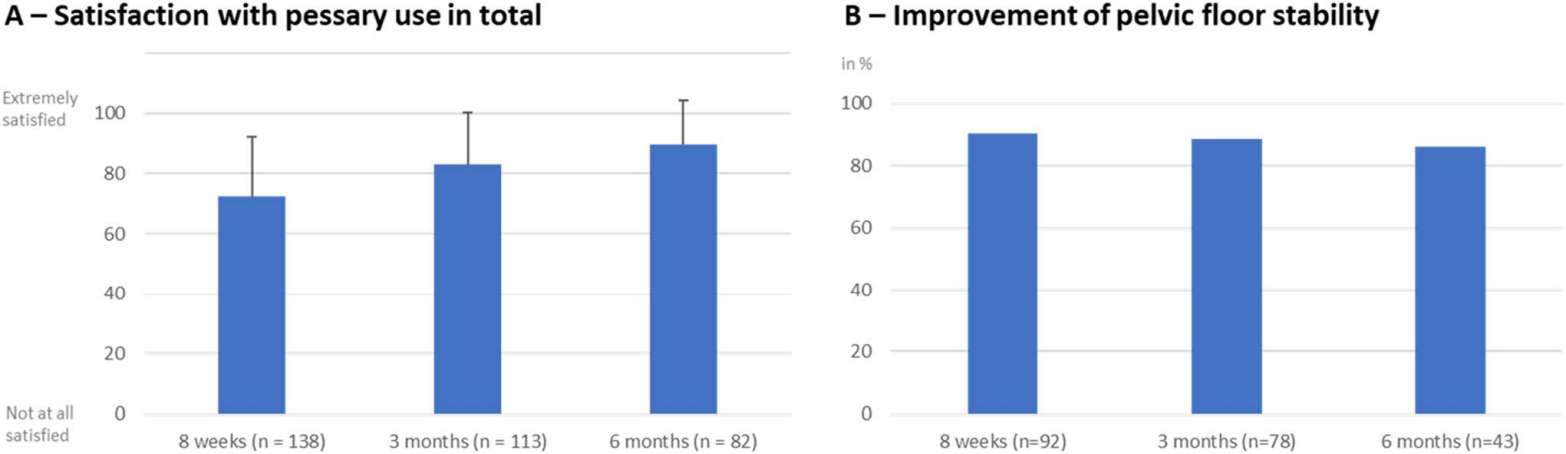
**A** Overall satisfaction with pessary use at the three time points on a scale from (not at all satisfied) – 100 (extremely satisfied). **B** Percentage of women feeling an improvement of pelvic floor stability at the three time points

Women without any pelvic floor disorders confirmed an increased sense of stability in the pelvic floor in 90% at 8 weeks, 88% at 3 months and 86% at 6 months (Fig. 4B).

The overall satisfaction on a scale from 0 (not satisfied at all) to 100 (extremely satisfied) was an average value of 73 at 8 weeks, 83 at 3 months and 90 at 6 months.

## Discussion

Pelvic floor disorders are common and associated with pregnancy and childbirth [1, 2]. Postpartum pelvic floor dysfunctions often originate from injuries of muscular, neurogenic and connective tissue structures [3–5]. Pelvic floor muscle training is recommended to women postpartum to address the muscular structures, although there is no respectively low evidence for the use in therapy and preventing urinary incontinence or POP [8–10]. To improve the nerve function after delivery, electrostimulation therapy can also be offered to women [11]. In orthopaedic disease patterns, early mobilisation and use of stabilising medical devices are often used for rehabilitation of the connective tissue [14]. Bihler et al. found a high interest in women using a postpartum pessary as a prevention strategy in their questionnaire study regarding the personal choice of mode of delivery [21]. For therapy of postpartum pelvic organ prolapse and stress urinary incontinence, the Restifem^®^ pessary is approved. It supports the anterior vaginal wall behind the symphysis, the lateral sulci and the sacro-uterine ligaments and therefore stabilises the connective tissue postpartum.

The aim of our prospective, multicentre study was to evaluate the willingness and compliance of postpartum women to support pelvic floor restitution using the Restifem^®^ pessary 6 weeks to 6 months after birth. Additionally, we focussed on applicability and arising problems. Furthermore, we determined the influence of the pessary use on symptoms of pelvic floor dysfunctions. So far, there are only two small pilot studies examining a pessary use in the postpartum period. One study revealed that 15 women with urinary incontinence stated to have an improvement of symptoms using a cube pessary [16]. The other study included 16 women and showed that ring pessary treatment early after delivery was feasible and seemed to reduce POP 6 weeks postpartum [15]. The authors considered the ring pessary form as possibly inadequate and stated that an anatomically adjusted form should be applied. For our study, we used the approved Restifem^®^ pessary, especially for postpartum women developed, which supports the vagina into an anatomical correct position by stabilising level I, level II and level III [17–19]. Two days after delivery and regardless of their delivery mode or parity, women were informed about the study. A high proportion of women (90%) was initially interested to take part and willing to use the pessary. We recruited women with and without subsequent pelvic floor disorders. Therefore, we were able to distinguish between the use of the pessary in a therapeutic as well as a stabilising respectively preventive approach. The response rate at the 8 weeks postpartum email survey was only 31%, and only 24% of questionnaires were answered adequately for further evaluation. Reason for this could be that the acceptance of the pessary use was low although there was an initial high interest. Perhaps, the information on possible advantages and application of the pessary 2 days after delivery to all women and the indiscriminate distribution was inappropriate. In addition, it is known that the reply rate in medical surveys via email are poorer compared to interviews in person or surveys by mail. A recent review found an average response rate in patients email surveys of 68–17% [22]. There might be other specific reasons, why women did not answer the questionnaire in our study. First, they used the pessary but found no time to fill out the survey due to the special situation with the newborn. Second, at some email providers, the automatically generated emails ended up in the spam folder, so only after the manual reminder, women were reached by email. A proportion of women was possibly not willing to take further part in our study. The fraction of inadequate answered questionnaires might be explained by the high number of questions. Nevertheless, 209 women at 8 weeks, 113 women at 3 months and 82 women at 6 months were included in the analysis (Fig. 2A). Initially, 34% of women did not want to use the pessary any further, the given reasons were discomfort and difficulties or time-consuming handling. Most of the women used the pessary daily especially in between 6 weeks and 3 months postpartum. The frequency of application and the proportion of women using the pessary decreased after 3 months. In the instruction manual and video, as well as in our study information, it is recommended to use the pessary for 3–6 months beginning after about 6 weeks postpartum and after wound healing is completed. This could be the explanation why women ended up using the application in the period between 3 and 6 months. There are several studies evaluating the compliance of pessary therapy in patients with POP, thereby the short-term compliance over about 1 year is estimated to be about 80% after successful fitting [23, 24]. In contrast to all available studies on pessary therapy, our study included mainly women without pelvic floor disorders and the pessary was not fitted by a health professional. Severe complications within pessary use are rare, but common side effects are dislodgment, discomfort or pain and de novo urinary symptoms [25]. In our study, none of the women stated that the pessary was too small, initially 25% of women felt the pessary too big. Overall, discomfort was reported in less than 10% and pain was rare, especially after 3 and 6 months (Table 1). Most women experience a certain feeling of pressure and the need for position correction in about 60% (Table 1). In total, the pessary seemed to be applicable without any severe complication or problems in most of our study participants.

Regarding the reduction of symptoms, most women stated to have no symptoms at all (Table 2). The interpretation of the data on pelvic floor disorders in our study should be viewed with caution. First, we did not use a validated questionnaire to estimate the symptoms and the number of women with symptoms was small. But the questionnaire contained clear questions about reduction of complaints. Especially women with POP stated to have an improvement in symptoms (Fig. 3). Women with bladder dysfunction also described an improvement, but a proportion has noticed a deterioration of symptoms (Fig. 3). In our study, we were not able to further differentiate which women had an improvement or deterioration. Pessary use in patients with POP can lead to symptoms of stress urinary incontinence [25]. In our opinion, stress urinary incontinence should be reduced by the Restifem^®^ pessary due to its level III support. Maybe, there are other bladder complaints increasing during Restifem^®^ use. Therefore, further evaluations are necessary.

In total, most women independent of pelvic floor symptoms felt a little to high improvement of pelvic floor stability and were highly satisfied using the pessary (Fig. 4A, B).

The main limitation of our study is the high initial dropout rate. The questionnaire was not validated, but to our knowledge, no suitable validated questionnaire concerning medical device therapy use exists. Additionally, we did not collect and correlate data on parity, age, delivery mode and further maternal characteristics.

The strength of the study was the prospective and multicentre design including an initially high number of participants. This is the first study to evaluate the willingness of women to use a pessary postpartum especially in a preventive and not only therapeutic approach. We were able to determine the major reasons why women were not willing to continue using the pessary and the main problems concerning pessary usage over time.

Taken together, the Restifem^®^ pessary is the first medical device established for enhancing the regeneration of the pelvic floor connective tissue after childbirth in a therapeutic and preventive approach. Women are willing to use of the Restifem^®^ pessary in the postpartum period and it is feasible and accompanied with less complications, primarily discomfort. Especially in women with pelvic floor disorders the use of the pessary reduced these symptoms, but also in women without symptoms, the pessary led to an increased sense of stability. Nevertheless, it does not seem to be sufficient to hand out the pessaries 2 days postpartum without any further personal instruction and supervision. At this point, midwifes and gynaecologists could help to inform and introduce women to improve the compliance and application.

## Conclusions

The Restifem^®^ pessary can be offered to women in the postpartum period to improve pelvic floor dysfunction and stability. Further prospective studies will be necessary to investigate whether the pessary use is appropriate to enhance the restitution of pelvic floor anatomy and function and thereby prevent pelvic floor disorders.

## Supplementary Information

Below is the link to the electronic supplementary material.

Supplementary file1 (DOCX 36 KB)

## Data Availability

All data associated with the manuscript and not included in tables, figures or supplemental material are available on request to the corresponding author.

## References

[1] Handa VL, Blomquist JL, Knoepp LR, Hoskey KA, McDermott KC, Muñoz A (2011). Pelvic floor disorders 5–10 years after vaginal or cesarean childbirth. Obstet Gynecol.

[2] Blomquist JL, Muñoz A, Carroll M, Handa VL (2018). Association of delivery mode with pelvic floor disorders after childbirth. JAMA.

[3] Allen RE, Hosker GL, Smith AR, Warrell DW (1990). Pelvic floor damage and childbirth: a neurophysiological study. Br J Obstet Gynaecol.

[4] Kruger JA, Budgett SC, Wong V, Nielsen PMF, Nash MP, Smalldridge J, Hayward LM, Tian TY, Taberner AJ (2017). Characterizing levator-ani muscle stiffness pre- and post-childbirth in European and Polynesian women in New Zealand: a pilot study. Acta Obstet Gynecol Scand.

[5] Dietz HP (2021). Ultrasound imaging of maternal birth trauma. Int Urogynecol J.

[6] Van de Waarsenburg MK, Verberne EA, van der Vaart CH, Withagen MIJ (2018). Recovery of puborectalis muscle after vaginal delivery: an ultrasound study. Ultrasound Obstet Gynecol.

[7] van Veelen GA, Schweitzer KJ, van der Vaart CH (2014). Ultrasound imaging of the pelvic floor: changes in anatomy during and after first pregnancy. Ultrasound Obstet Gynecol.

[8] Woodley SJ, Lawrenson P, Boyle R, Cody JD, Mørkved S, Kernohan A, Hay-Smith EJC (2020). Pelvic floor muscle training for preventing and treating urinary and faecal incontinence in antenatal and postnatal women. Cochrane Database Syst Rev.

[9] Bø K, Anglès-Acedo S, Batra A, Brækken IH, Chan YL, Jorge CH, Kruger J, Yadav M, Dumoulin C (2022). International urogynecology consultation chapter 3 committee 2; conservative treatment of patient with pelvic organ prolapse: pelvic floor muscle training. Int Urogynecol J.

[10] Schütze S, Heinloth M, Uhde M, Schütze J, Hüner B, Janni W, Deniz M (2022). The effect of pelvic floor muscle training on pelvic floor function and sexuality postpartum. A randomized study including 300 primiparous. Arch Gynecol Obstet.

[11] Alouini S, Memic S, Couillandre A (2022). Pelvic floor muscle training for urinary incontinence with or without biofeedback or electrostimulation in women: a systematic review. Int J Environ Res Public Health.

[12] Bodner-Adler B, Bodner K, Stinglmeier A, Kimberger O, Halpern K, Koelbl H, Umek W (2019). Prolapse surgery versus vaginal pessary in women with symptomatic pelvic organ prolapse: which factors influence the choice of treatment?. Arch Gynecol Obstet.

[13] Al-Shaikh G, Syed S, Osman S, Bogis A, Al-Badr A (2018). Pessary use in stress urinary incontinence: a review of advantages, complications, patient satisfaction, and quality of life. Int J Womens Health.

[14] Karlsson J, Eriksson BI, Swärd L (1996). Early functional treatment for acute ligament injuries of the ankle joint. Scand J Med Sci Sports.

[15] Baessler K, Heihoff-Klose A, Boelke S, Stupin J, Junginger B (2019). Does an early postpartum pessary treatment lead to remission of pelvic organ prolapse after vaginal birth? A pilot study. Int Urogynecol J.

[16] Lange R, Tabibi E, Hitschold T, Lange S, Naumann G (2021). Beckenboden-REhabilitations-STudie BREST. Kongress der Deutschen Kontinenz Gesellschaft sine loco [digital], 05.-06.-11.2021.

[17] DeLancey JO (1992). Anatomic aspects of vaginal eversion after hysterectomy. Am J Obstet Gynecol.

[18] Huebner M, DeLancey JOL (2019). Levels of pelvic floor support: what do they look like on magnetic resonance imaging?. Int Urogynecol J.

[19] Beilecke K (2019). Postpartale Beeinflussung des Beckenbodenbindegewebes—ein bisher vernachlässigter Therapieansatz. Frauenarzt.

[20] Baessler K, Junginger B (2011). Beckenboden-Fragebogen für Frauen. Validierung eines Instrumentes mit posttherapeutischem Modul zur Evaluation von Symptomen, Leidensdruck, Lebensqualität, Verbesserung und Zufriedenheit. Aktuelle Urol.

[21] Bihler J, Tunn R, Reisenauer C, Kolenic GE, Pauluschke-Froehlich J, Wagner P, Abele H, Rall KK, Naumann G, Wallwiener S, Wallwiener M, Sohn C, Brucker SY, Huebner M (2019). The preferred mode of delivery of medical professionals and non-medical professional mothers-to-be and the impact of additional information on their decision: an online questionnaire cohort study. Arch Gynecol Obstet.

[22] Meyer VM, Benjamens S, Moumni ME, Lange JFM, Pol RA (2022). Global overview of response rates in patient and health care professional surveys in surgery: a systematic review. Ann Surg.

[23] Hsieh MF, Tsai HW, Liou WS, Lo CC, Lin ZH, An YF, Lin HY (2019). Long-term compliance of vaginal pessaries: does stress urinary incontinence matter?. Medicine (Baltimore).

[24] Manchana T (2011). Ring pessary for all pelvic organ prolapse. Arch Gynecol Obstet.

[25] Manzini C, Morsinkhof LM, van der Vaart CH, Withagen MIJ, Grob ATM (2022). Parameters associated with unsuccessful pessary fitting for pelvic organ prolapse up to three months follow-up: a systematic review and meta-analysis. Int Urogynecol J.

