# Supplementary material for: Evaluating compliance and applicability of postpartum pessary use for preventing and treating pelvic floor dysfunction: a prospective multicenter study
Source: Arch Gynecol Obstet. 2023 May 21;308(2):651–9. doi: 10.1007/s00404-023-07075-9 (PMC10293351; doi:10.1007/s00404-023-07075-9)
Supplement: Supplementary file 1 — Supplementary file1 (DOCX 36 KB) [file 404_2023_7075_MOESM1_ESM.docx]

**Supplemental Figure 1: Questionnaire Pessary Compliance**

**General**

| Are you still wearing your pessary? | 🗆 yes | 🗆 no |  |
| --- | --- | --- | --- |
| If not, why? | 🗆 Pessary uncomfortable | 🗆 Pessary hurts | 🗆Urination not possible with pessary |
|  | 🗆Defecation not possible with pessary | 🗆Handling is too difficult | 🗆Time-consuming |
|  | 🗆Other (comment) |  |  |
| Are you breastfeeding? | 🗆 no |  | 🗆 yes |
| Have your birth injuries healed? | 🗆 had none | 🗆 yes | 🗆 no |
| Do you have pain in the genital area? | 🗆 no |  | 🗆 yes |
| Is the pessary the right size? | 🗆 yes | 🗆 no – to big | 🗆 no – to small |

**Pessary application**

| Are the operating instructions comprehensible? | 🗆 yes |  |  | 🗆 no |
| --- | --- | --- | --- | --- |
| Is the video of instructions comprehensible? | 🗆 yes |  |  | 🗆 no |
| The insertion of the pessary is … | 🗆 very easy | 🗆 fairly easy | 🗆 quite difficult | 🗆 very hard |
| Do you wet he pessary with water to be able to insert it? | 🗆 no |  |  | 🗆 yes |
| Do you wet he pessary with vaseline/lubricating cream to be able to insert it? | 🗆 no | 🗆 yes, with vaseline | 🗆 yes, with lubricant | 🗆 yes, with hormone creme |
|  | 🗆 other (comment) |  |  |  |
| Is it necessary to correct the position of the pessary? | 🗆 never | 🗆 while walking | 🗆 when sitting | 🗆 at rest |
|  |  | 🗆 after defecation | 🗆 after urination | 🗆 when changing position e.g., sitting down, bending down? |
| Is there a feeling of pressure while wearing the pessary? | 🗆 never | 🗆 while walking | 🗆 when sitting | 🗆 at rest |
|  |  | 🗆 after defecation | 🗆 after urination | 🗆 when changing position e.g., sitting down, bending down? |
| Is it possible to urinate while wearing the pessary? | 🗆 yes |  |  | 🗆 no |
| Is defecation possible while wearing the pessary? | 🗆 yes |  |  | 🗆 no |
| Does the pessary produce increased discharge that bothers you? | 🗆 no |  |  | 🗆 yes |
| Does the wearing comfort decrease? | 🗆 never | 🗆 after 4 hours | 🗆 after 8 hours | 🗆 after 12 hours |
| Removing the pessary is … | 🗆 very easy | 🗆 fairly easy | 🗆 quite difficult | 🗆 very hard |

**Symptom relief**

| If you have problems with pelvic organ prolapse, does wearing the pessary reduce them? | 🗆 not applicable – have no symptoms | 🗆 not at all | 🗆 a little | 🗆 quite | 🗆 very |
| --- | --- | --- | --- | --- | --- |
| If you have problems with urinary incontinence, do they decrease while wearing the pessary? | 🗆 not applicable – have no symptoms | 🗆 not at all | 🗆 a little | 🗆 quite | 🗆 very |
| If you have increased or premature urge to urinate, does wearing the pessary reduce it? | 🗆 not applicable – have no symptoms | 🗆 not at all | 🗆 a little | 🗆 quite | 🗆 very |
| If you have no complaints: Did wearing the pessary improve the feeling of stability in the pelvic area? | 🗆 very | 🗆 quite | 🗆 a little | 🗆 not at all | 🗆 it got worse |

**Compliance**

| Are you able to manage the pessary without contact with your midwife/doctor? | 🗆 yes |  |  | 🗆 no |
| --- | --- | --- | --- | --- |
| Do you have more genital infections? | 🗆 no |  |  | 🗆 yes |
| How often do you wear the pessary? | 🗆 daily – more than 8 hours | 🗆 daily – less then 8 hours | 🗆 sometimes | 🗆 never |
| If not, why? | 🗆 Pessary uncomfortable | 🗆 Pessary hurts | 🗆Urination not possible with pessary | 🗆Defecation not possible with pessary |
|  | 🗆Handling is too difficult | 🗆Time-consuming | 🗆not wearing during menstruation |  |
| The effort using the pessary is… | 🗆 low | 🗆 moderate | 🗆 high | 🗆 to high |
| Do you like wearing the pessary? | 🗆 yes - always | 🗆 most of he time | 🗆 sometimes | 🗆 no -never |
| If not, why? | 🗆 Pessary uncomfortable | 🗆 Pessary hurts | 🗆Urination not possible with pessary | 🗆Defecation not possible with pessary |
|  | 🗆Handling is too difficult | 🗆Time-consuming | 🗆not wearing during menstruation | 🗆other (comment) |

**Overall satisfaction**

| How would you assess your bladder function using the pessary? | 🗆 not applicable – have no dysfunction | 🗆 great improvement | 🗆 slight improvement | 🗆 no change | 🗆 some deterioration | 🗆 great deterioration |
| --- | --- | --- | --- | --- | --- | --- |
| How would you assess your bowl function using the pessary? | 🗆 not applicable – have no dysfunction | 🗆 great improvement | 🗆 slight improvement | 🗆 no change | 🗆 some deterioration | 🗆 great deterioration |
| How would you assess your descensus using the pessary? | 🗆 not applicable – have no dysfunction | 🗆 great improvement | 🗆 slight improvement | 🗆 no change | 🗆 some deterioration | 🗆 great deterioration |
| How would you rate your sexual function using the pessary? | 🗆 not applicable – have no dysfunction | 🗆 great improvement | 🗆 slight improvement | 🗆 no change | 🗆 some deterioration | 🗆 great deterioration |

How satisfied are you with the achieved result of the therapy?

Please mark the digit that applies to you on the scale from 0 to 100.

Not at all satisfied Extremely satisfied

_______________________________________________________________________________

0 50 100
